# *Ocimum gratissimum* Aqueous Extract Protects H9c2 Myocardiac Cells from H_2_O_2_-Induced Cell Apoptosis through Akt Signalling

**DOI:** 10.1155/2011/578060

**Published:** 2010-08-30

**Authors:** Mu-Jang Lee, Han-Min Chen, Bor-Show Tzang, Chiu-Wen Lin, Chau-Jong Wang, Jer-Yuh Liu, Shao-Hsuan Kao

**Affiliations:** ^1^Department of Internal Medicine, Division of Cardiology, Tian-Sheng Memorial Hospital, Pingtung 92843, Taiwan; ^2^Department of Life Science, Fu-Jen Catholic University, Taipei 24205, Taiwan; ^3^Institute of Biochemistry and Biotechnology, Chung Shan Medical University, Taichung 40201, Taiwan; ^4^Graduate Institute of Cancer Biology, China Medical University, Taichung 40402, Taiwan; ^5^Center for Molecular Medicine, China Medical University Hospital, Taichung 40402, Taiwan; ^6^Clinical Laboratory, Chung Shan Medical University Hospital, Taichung 40201, Taiwan

## Abstract

Increased cell death of cardiomyocyte by oxidative stress is known to cause dysfunction of the heart. *O. gratissimum* is one of the more well-known medicinal plants among the *Ocimum* species and widely used in treatment of inflammatory diseases. In this study, we hypothesized that aqueous extract of *O. gratissimum* leaf (OGE) may protect myocardiac cell H9c2 from oxidative injury by hydrogen peroxide (H_2_O_2_). Our results revealed that OGE pretreatment dose-dependently protects H9c2 cells from cell death when exposed to H_2_O_2_. Additionally, DNA condensation induced by H_2_O_2_ was also reduced by OGE pretreatment, suggesting that *Ocimum gratissimum* extract may attenuate H_2_O_2_-induced chromosome damage. Further investigation showed that OGE pretreatment inhibited H_2_O_2_-induced activation of caspase-3 and caspase-9, as well as H_2_O_2_-induced upregulation of proapoptotic Apaf-1 and the release of cytosolic cytochrome c, but has little effect on the activation of caspase-8. Additionally, OGE pretreatment significantly upregulated Bcl-2 expression and Akt phosphorylation, and slightly affected the phosphorylation of mitogen-activated protein kinases including p38 MAPK and JNK. Taken together, our findings revealed that *Ocimum gratissimum* extract effectively inhibited the mitochondrial pathway and upregulated Bcl-2 expression, which may be important in protecting H9c2 cells from H_2_O_2_-induced cell death.

## 1. Introduction

It is known that cardiac cell apoptosis, a result of oxidative stress by ischemia and reperfusion, plays an important role in the pathogenesis of heart dysfunctions [1–4]. Oxidative stress-induced reactive oxygen species (ROS) increase membrane lipid peroxidation and open voltage-sensitive Ca^2+^ channels or Na^+^/Ca^2+^ exchangers in vascular tissues during ischemia and reperfusion (I/R), resulting in extracellular Ca^2+^ influx-related heart failure [5–8]. The accumulation of intracellular Ca^2+^ alters the mitochondrial membrane permeability which leads to the release of cytochrome c into the cytoplasm and the following apoptotic cascades [5, 9, 10]. The released cytochrome c then combines with apoptosis protease-activating factor-1 (Apaf-1) and pro-caspase-9 and forms an intermediary complex apoptosome which activates caspace-3 and causes mitochondrial apoptosis [9, 11].

It has been proposed that the appearance of ROS are related to the activation of mitogen-activated protein kinases (MAPK) such as the p38 MAPK (p38) and the c-Jun N-terminal kinase (JNK). These two kinases cause the phosphorylation and translocation of nuclear factor-*κ*B (NF-*κ*B) which in turn leads to the synthesis and release of tumor necrosis factor-*α* (TNF-*α*) and the initiation of a death receptor-dependent apoptotic pathway [11–14]. Therefore, ROS are regarded as important factors in the pathogenesis of myocardial I/R injury for its induction of apoptosis of myocardiac cells through two known pathways—a mitochondrial pathway and a death receptor-mediated pathway.

*Ocimum gratissimum* is widely distributed in tropical and warm temperate geolocations and commonly used in folk medicine [15, 16]. Evaluation of its biological activities revealed that *Ocimum gratissimum'*s abundant antioxidant content allows it many therapeutic functions, including anti-inflammation [17], analgesic and spasmolytic activities [18], antidiarrheal activity [16], antitumor activity [19], antiviral activity [20], and antihyperglycemic activity [21], and the improvement of the phagocytic function without affecting the humoral or cell-mediated immune system [22]. Therefore, it is suggested that *Ocimum gratissimum* is a suitable candidate for the treatment of oxidative stress-induced disorders.

In this study, we aimed to examine the effects of aqueous *Ocimum gratissimum* leaf extract (OGE) on hydrogen peroxide (H_2_O_2_)-treated H9c2 myocardiac cells and investigate the protective mechanisms of *Ocimum gratissimum*.

## 2. Materials and Methods

### 2.1. Chemicals

Aprotinin, leupeptin, hydrogen peroxide (H_2_O_2_), 3-(4,5-dimethylthiazol-2-yl)-2,5-diphenyl-tetrazolium bromide (MTT), 4,6-diamidino-2-phenylindole dihydrochloride (DAPI), Nonidet P-40, phenylmethylsulfonyl fluoride (PMSF), sodium fluoride, sodium chloride, sodium phosphate, Tris-HCl, and Tween-20 were purchased from Sigma-Aldrich (St. Louis, MO, USA).

### 2.2. Preparation of OGE and Composition Analysis

Leaves of *Ocimum gratissimum* Linn were harvested, washed with distilled water, and then homogenized with distilled water by using polytron. The homogenate was incubated at 95°C for 1 hour (h) and then filtered through two layers of gauze. The filtrate was centrifuged to remove insoluble pellets (20,000 g for 15 min at 4°C) and the supernatant (OGE) was collected, lyophilized, and stored at −70°C until use.

The content of polyphenol in OGE was analyzed as indicated in a previous paper [23], revealing the final extract (OGE) composition of 11.1% polyphenolic acid and 4.5% flavonoids.

### 2.3. Cell Culture and Experimental Treatments

The myocardiac cells H9c2 were obtained from American Type Culture Collection (ATCC; Rockville, MD) and maintained in Dulbecco's modified Eagle's medium supplemented with 10% v/v fetal bovine serum (Gibco BRL, Gaithersburg, MD, USA) and 100 *μ*g/mL penicillin/streptomycin (Sigma-Aldrich Chemie, Munich, Germany) at 37°C in a humidified atmosphere containing 5% CO_2_. In all conditions, H9c2 cells were seeded in 6-well culture plates at an initial density of 1 × 10^5^ cells/mL and grown to approximately 80% confluence. Oxidative stress was induced by treating with freshly prepared H_2_O_2_. Cells were pretreated with OGE at indicated concentrations for 3 hours (hrs), and then the medium containing H_2_O_2_ was added (final concentration at 200 *μ*M) and incubated for indicated amounts of time. After the incubation, the cells were washed with phosphate-buffered saline (PBS; 25 mM sodium phosphate, 150 mM NaCl, pH 7.2) and then collected for the subsequent analysis.

### 2.4. MTT Assay for Cell Viability

Cell viability was determined by MTT assay. H9c2 cells were pretreated with 0, 50, and 100 *μ*g/mL OGE for 3 hrs, and then treated with 200 *μ*M H_2_O_2_ for 24 hrs. After the 24 hrs treatments, medium was removed, and the H9c2 cells were incubated with MTT (0.5 mg/mL) at 37°C for 4 hrs. The viable cell number was directly proportional to the production of formazan, which was dissolved in isopropanol and determined by measuring the absorbance at 570 nm using a microplate reader (SpectraMAX 360 pc, Molecular Devices, Sunnyvale, CA).

### 2.5. DAPI Staining

H9c2 cells (5 × 10^4^ cells/mL) were pretreated with 0, 50, and 100 *μ*g/mL OGE for 3 hours (h) and then incubated with 200 *μ*M H_2_O_2_ for 3 hrs. After the treatment, the cells were stained with DAPI and photographed using a fluorescence microscope as previously described [24, 25]. The incidence of DNA condensation in each preparation was analyzed by counting 300 cells and determining the percentage of DNA condensed-cells [26].

### 2.6. Immunoblotting

H9c2 cells (5 × 10^4^ cells/mL) were pretreated with 0, 50, and 100 *μ*g/mL OGE for 3 hrs and then incubated with 200 *μ*M H_2_O_2_ for 3 hrs. For cytosolic cytochrome c analysis, the treated H9c2 cells were washed with PBS and homogenized in 0.25 M ice-cold sucrose solution containing 1 mM PMSF, 1 mM sodium fluoride, and 10 *μ*g/mL aprotinin and leupeptin at 4°C. The homogenate was subjected to differential centrifugation and the different fractions were separated as follows: structural proteins, nucleus, and cell debris at 600× g for 10 min; mitochondria at 5000× g for 10 min; lysosomes at 15,000× g for 10 min; microsomes at 1,20,000× g for 30 min and the supernatant, cytosol. The protein concentration of cytosolic fraction was determined by using Bradford method (Bio-Rad Laboratory (Hercules, CA, USA)).

For total cell lysate, the treated H9c2 cells were washed with PBS and lysed in a lysis buffer (50 mM Tris-HCl, pH 7.5, 150 mM NaCl, 1% Nonidet P-40, 1 mM PMSF, 1 mM sodium fluoride, and 10 *μ*g/mL aprotinin and leupeptin). The lysates were incubated on ice for 30 min and centrifuged at 20,000 g for 15 min. The supernatants were collected and followed by protein quantitation using Bradford method (Bio-Rad).

Crude proteins (30 *μ*g per lane) were electrophoresed on 12.5% SDS-polyacrylamide gel, and transferred onto a nitrocellulose membrane (Millipore, Bedford, MA) as previously described [27]. The blotted membrane was blocked with 5% w/v skimmed milk in PBS, and then incubated for 2 hrs with 1/1000 dilution of the specific antibodies against human caspase-3, caspase-9, Apaf-1, cytochrome c (CytC), Bcl-2, truncated Bid (tBid), phosphorylated ERK1/2 (p-ERK1/2), phosphorylated-JNK (p-JNK), phosphorylated p38 (p-p38), phosphorylated Akt (p-Akt) (Cell Signaling Technologies, Beverly, MA), and *β*-actin (Abcam Inc., Cambridge, UK). Bound antibodies were detected using 1/2000 dilution of peroxidase-conjugated secondary antibodies (Abcam Inc., Cambridge, UK) and ECL chemiluminescence reagent (Millipore, Bedford, MA) as the substrate system. Quantitative analysis was performed by densitometry.

### 2.7. Statistical Analysis

Data were expressed as means ± SEMs of the three independent experiments. Statistical significance analysis was determined by using 1-way ANOVA followed by Dunnett for multiple comparisons with the control. The differences were considered significant for *P* values less than .05.

## 3. Results

### 3.1. Effect of OGE on H_2_O_2_-Induced Cell Death in H9c2 Cells

**Figure 1:**
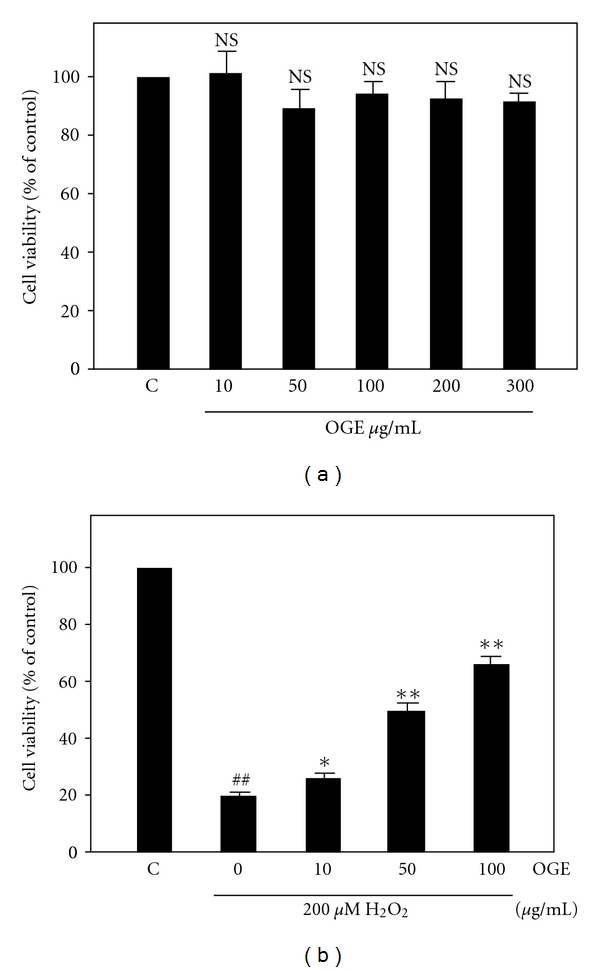
Effects on cell viability of H9c2. (a) The cell viability of H9c2 cells treated with a series concentration of OGE (10, 50, 100, 200, and 300 *μ*g/mL) for 24 hrs. (b) The cell viability of H9c2 cells pretreated with a series concentration of OGE (10, 50, and 100 *μ*g/mL) for 3 hrs and then treated with 200 *μ*M of H_2_O_2_ for 24 hrs. Three independent experiments were performed for statistical analysis. NS, not significant; ##, *P* < .01 as comparing to control labeled “C”; ∗, *P* < .05 and ∗∗, *P* < .01 as comparing to 0 *μ*g/mL of OGE.

Prior to investigating whether *Ocimum gratissimum* extract protected H9c2 cells from H_2_O_2_-induced cell death, the cytotoxicity of the extract was determined by dividing the H9c2 cells into dishes and treated with different dosages of OGE (10, 50, 100, 200, and 300 *μ*g/mL) for 24 hrs, and performing an MTT assay for cell viability. As shown in Figure 1(a), although the cell viability appeared to be slightly decreased by the different strengths of OGE treatments, the changes in cell viability were not statistically significant when compared to the control group. To examine the effects of OGE on H_2_O_2_-induced cell death, the cell viability of H9c2 cells pretreated with 0, 10, 50, and 100 *μ*g/mL OGE and then treated with 200 *μ*M H_2_O_2_ was determined. As shown in Figure 1(b), H_2_O_2_ treatment greatly diminished the cell viability to 18.7 ± 0.6% of control. Interestingly, OGE pretreatment (10, 50, and 100 *μ*g/mL) before H_2_O_2_ treatment significantly rose the cell viability to 25.3 ± 0.8%, 50.6 ± 2.7% and 68.6 ± 3.8% of control, respectively. Moreover, the various concentrations (10, 50, 100 *μ*g/mL) of OGE alone did not affect cell viability (data not shown). These findings indicate that OGE pretreatment dose-dependently increased the cell viability diminished by H_2_O_2_ treatment, suggesting that OGE is capable of protecting H9c2 cell from H_2_O_2_-induced cell death.

### 3.2. Inhibition of H_2_O_2_-Induced DNA Condensation by OGE in H9c2 Cells

**Figure 2:**
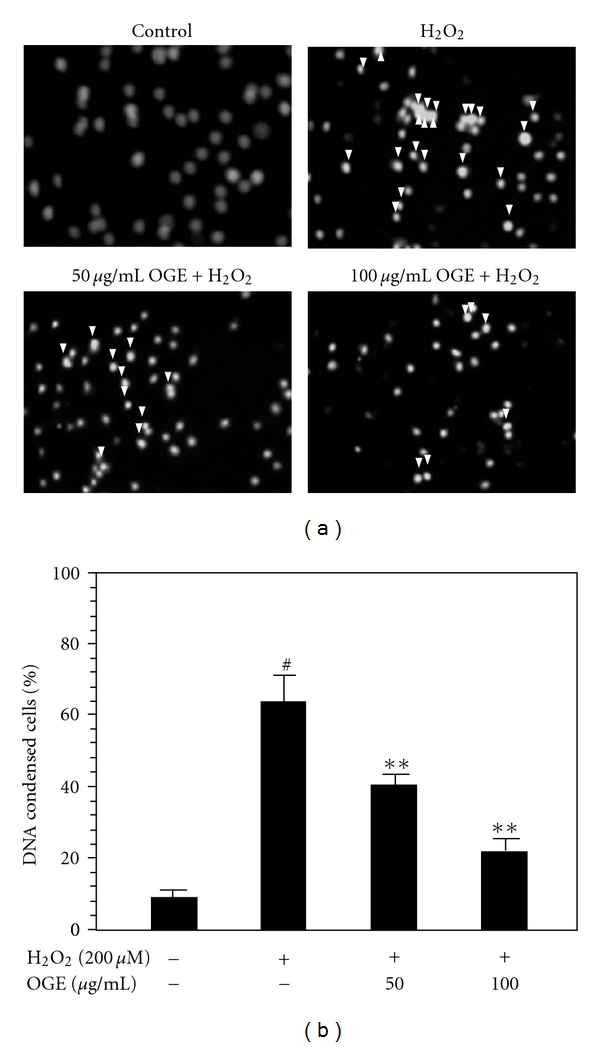
Inhibition of the H_2_O_2_-induced DNA condensation. (a) H9c2 cells were pretreated with 0, 50, and 100 *μ*g/mL OGE for 3 and then treated with H_2_O_2_ for 3 hrs. After the treatments, the H9c2 cells were DAPI stained and photographed by fluorescence microscopy (200x). The cells which showed DNA condensation are indicated by arrow. (b) Incidence of DNA condensation of H9c2 cells. Data is presented as mean ± S.D., *n* = 3; #, *P* < .01 compared with control; ∗∗, *P* < .01 compared with H_2_O_2_ treatment alone.

To further investigate the H_2_O_2_-induced cell death and the protective effects of OGE, DNA condensation was monitored by DAPI staining. As shown in Figure 2(a), H9c2 cells treated with H_2_O_2_ increase the incidence of DNA condensation to 64.2 ± 6.1%, suggesting that H_2_O_2_ treatment may induce DNA condensation. Pretreatment with 50 and 100 *μ*g/mL OGE dose-dependently attenuated the incidence of DNA condensation of H9c2 cells to 40.7 ± 2.8% and 22.7 ± 2.4%, respectively. These findings indicate that H_2_O_2_-induced DNA condensation in H9c2 cells could be significantly reduced by OGE pretreatment. It is suggested that OGE pretreatment may alleviate the H_2_O_2_-induced DNA condensation and the subsequent cell damage.

### 3.3. Effect of OGE on H_2_O_2_-Induced Apoptosis in H9c2 Cells

**Figure 3:**
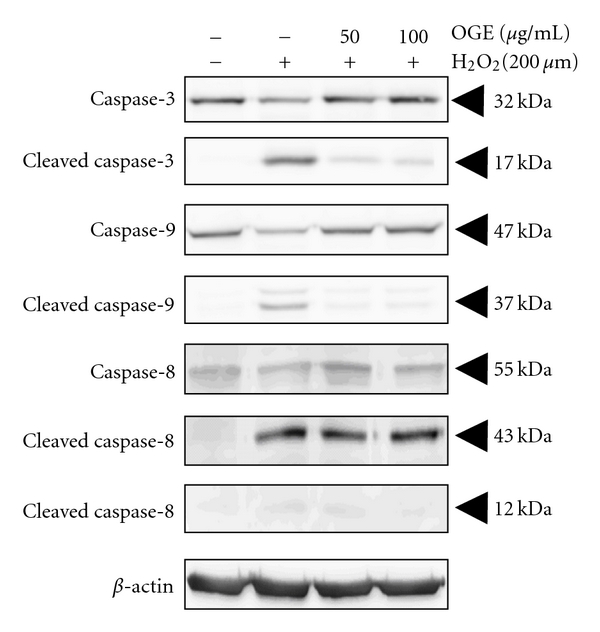
Protective effects via modulating the mitochondrial pathway. H9c2 cells were pretreated with 0, 50, and 100 *μ*g/mL OGE for 3 hrs and then treated with H_2_O_2_ for 3 hrs. After the treatments, the H9c2 cells were lyzed for immunoblotting. The protein levels of caspase-3, cleaved caspase-3, caspase-9, cleaved caspase-9, caspase-8, and cleaved caspase-8 are, respectively, indicated.   *β*-actin was used for normalization.

DNA condensation is one of the characters of apoptosis; therefore, the effects of *Ocimum gratissimum* extract on the intrinsic/mitochondrial pathway (through CytC, caspase-3, caspase-9) and the extrinsic pathway (through caspase-8) were investigated. As shown in Figure 3, H_2_O_2_ treatment diminished the level of caspase-3 (precursor form, 32 kDa), and increased the level of cleaved caspase-3 (active form, 17 kDa), cleaved caspase-9 (active form, 37 kDa), and cleaved caspase-8 (active form, 43 kDa). Interestingly, pretreatment with *Ocimum gratissimum* extract reduced the level of cleaved caspase-3 and cleaved caspase-9, but only slightly affected the level of cleaved caspase-8, suggesting that OGE's protective effects mainly influence the mitochondrial pathway.

**Figure 4:**
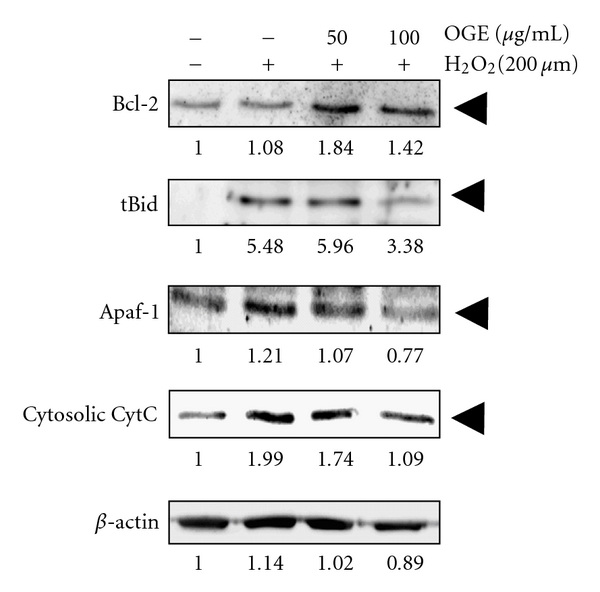
Effects of *Ocimum gratissimum* extracts on protein level of Bcl-2, tBid, Apaf-1, and Cytosolic cytochrome c (CytC). H9c2 cells were pretreated with 0, 50, and 100 *μ*g/mL OGE for 3 hrs and then treated with H_2_O_2_ for 24 hrs. After the treatments, the H9c2 cells were lyzed for immunoblotting. The protein levels of Bcl-2, Apaf-1, and cytochrome c are, respectively, indicated. *β*-actin was used for normalization.

To further investigate the effects of *Ocimum gratissimum* extract on mitochondrial pathway, the level of upstream mediators, including Apaf-1, cytosolic CytC, tBid, and Bcl-2 were compared. As shown in Figure 4, after normalizing with corresponding level of *β*-actin, H_2_O_2_ treatment alone showed a slight decrease in the level of Bcl-2 to 0.95 fold of control but a large increase in the level of tBid, Apaf-1 and cytosolic CytC, the coactivators of apoptosome for the activation of caspase-9, to 4.81, 1.06, and 1.75 fold of control, respectively. Additionally, OGE pretreatment (100 *μ*g/mL) increased the level of Bcl-2 to 1.68 fold of H_2_O_2_ treatment alone and diminished the levels of tBid, Apaf-1, and cytosolic CytC to 0.79, 0.82, and 0.71 fold of H_2_O_2_ treatment alone, respectively. Moreover, the treatment of OGE alone showed no significant effect on the levels of the tested caspases and apoptotic components in H9c2 cell (data not shown). Taken together, these findings indicate that H_2_O_2_ treatment may cause the cell death of H9c2 by inducing apoptosis, which may be attenuated by OGE pretreatment through inhibition of the mitochondrial pathway.

### 3.4. Effect of OGE on H_2_O_2_-Induced Akt and MAPK Activation in H9c2 Cells

**Figure 5:**
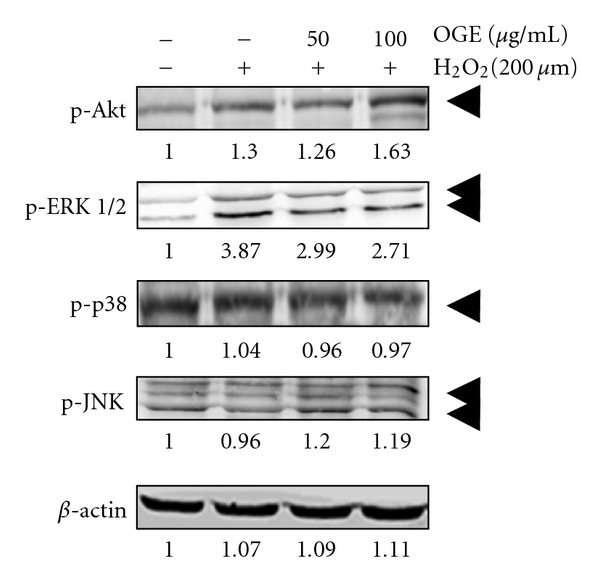
Effects of *Ocimum gratissimum* extracts on the activatation of Akt and MAPKs. H9c2 cells were pretreated with 0, 50, and 100 *μ*g/mL OGE for 3 hrs and then treated with H_2_O_2_ for 3 hrs. After the treatments, the H9c2 cells were lyzed for immunoblotting. The phosphorylation of Akt (p-Akt), ERK1/2 (p-ERK), p38 (p-p38), and JNK (p-JNK) are, respectively, indicated*.β*-actin was used for normalization.

MAPKs and phosphatidylinositol 3-kinase/Akt signal cascades have been demonstrated to play important roles in both apoptosis and cell survival [28]. Therefore, the effects of *Ocimum gratissimum* extract on MAPKs and Akt activation were investigated. As shown in Figure 5, H_2_O_2_ treatment enhanced the phosphorylation of Akt (pAkt) and ERK 1/2 (p-ERK 1/2) to 1.30 fold and 3.87 fold, respectively, as compared to the control. Interestingly, 100 *μ*g/mL OGE pretreatment also increased the phosphorylation of Akt (to 1.63 fold) as compared to the control, but decreased the phosphorylation of ERK 1/2 to 0.69 fold as compared to H_2_O_2_ treatment alone. Additionally, the phosphorylation of p38 MAPK (p-p38) and JNK (p-JNK) was affected by neither H_2_O_2_ treatment alone nor OGE pretreatment. Additionally, the treatment of OGE alone increased the phosphorylation of Akt, but showed no significant effect on the phosphorylation of the other tested kinases in H9c2 cell (Supplement figure 1). Taken together, these findings suggest that *Ocimum gratissimum* extract may attenuate H_2_O_2_-induced cell death through the enhancement of the Akt-mediated survival signaling, and also implicate that there is a relationship between ERK1/2 activation and H_2_O_2_-induced cell apoptosis.

## 4. Discussion

Direct exposure of cells with oxidants, such as H_2_O_2_, was thought to always cause necrosis. But recent studies have shown that ROS can also induce cellular senescence and apoptosis under certain conditions [29, 30]. Our results reveal that H_2_O_2_ treatment alone significantly diminishes the viability of cells to 18.7 ± 0.6% of control and pretreatment with *Ocimum gratissimum* extract helps to increase cell viability to 68.6 ± 3.8% of control, suggesting that the extract is capable of protecting H9c2 cells from H_2_O_2_-induced cell death.

The mitochondrion is an important target of ROS, and interaction of mitochondria and ROS usually leads to the dysfunction of mitochondria and the subsequent apoptotic cascades [31]. *In situ* generated ROS can open the permeability transition (PT) pore and lead to subsequent changes in mitochondrial membrane potential which cause CytC release into the cytosol. Cytosolic CytC is required for the formation of the apoptosome and the resulting activation of procaspase-9 which in turn cleaves and activates the downstream effecter caspase-3 which then leads to eventual cell apoptosis [32, 33]. On the other hand, the increase in the expression of apoptosis-inhibitory proto-oncogene products such as Bcl-2 can inhibit the ROS induced mitochondrial PT pore from opening [34] and prevent the sequence of CytC release and cell apoptosis. Our findings reveal that OGE pretreatment increased the level of Bcl-2, suggesting that OGE's protective effects consists of the inhibition of the CytC release and apoptosome formation, resulting in the inhibition of mitochondrial pathway activation.

Furthermore, activated caspase-8 is reported to cleave the Bid protein to form tBid which subsequently enhances CytC release [35, 36]. Our findings reveal that although OGE pretreatment does not significantly decrease the activation of H_2_O_2_-induced caspase-8, it does diminish the formation of tBid. This finding therefore indicates that *Ocimum gratissimum* extract does not attenuate caspase-8 activation but may modulate the cleavage of Bid and alleviate apoptosis through other mechanisms.

Polyphenols from plant extracts have been demonstrated as being major therapeutic components for oxidative stress. Although the cellular mechanisms underlying the actions of polyphenols and their metabolites have not been completely interpreted, it is believed that their properties which allow antioxidant activity, free radical scavenging, and MAPK signaling, pathway targeting should be involved [37, 38]. The MAPK family, comprising of ERKs, JNK, and p38, is activated in response to the various stress stimuli caused by virus infections or chemical exposures. Previous studies have shown that early activation of ERK1/2 blocks caspase-3 activation through the repression of CytC release, and that late activation of ERK1/2 has significantly less effect on the repression of CytC release [39, 40]. ERK activation is also reported to play an essential role in cisplatin-induced CytC release and subsequent capase-3 activation [41]. Our results reveal that H_2_O_2_ treatment alone yielded higher ERK1/2 phosphorylation level, while OGE pretreatment yielded lower ERK1/2 phosphorylation levels. This implicates that *Ocimum gratissimum* extract facilitated the early activation of ERK1/2, and the data from the OGE treatment shows that ERK1/2 levels in the late stages were being restored to their original level. However, the detailed mechanisms need further investigation.

Many factors regulate cell survival through the phosphatidylinositol 3-kinase (PI3K)/Akt pathway [42]. It is known that activated PI3K/Akt promotes cell survival via the direct regulation of antiapoptotic Bcl-2 and apoptotic proteins including BAD, BCL-X_L_, and caspase-9 [43, 44], and that PI3K/Akt pathway is also involved in protecting skeletal muscle cells against oxidative damage [45]. Our findings show that when comparing H_2_O_2_ treatment only and OGE pretreatment (100 *μ*g/mL), the p-Akt is increased to 1.30- and 1.63-fold of control, respectively. It is suggested that oxidative stress may induce Akt activation, which may be due to the cell's own survival mechanism, and *Ocimum gratissimum* extract may further enhance the Akt-mediated survival signaling by the upregulation of Bcl-2 to alleviate cell injury.

**Figure 6:**
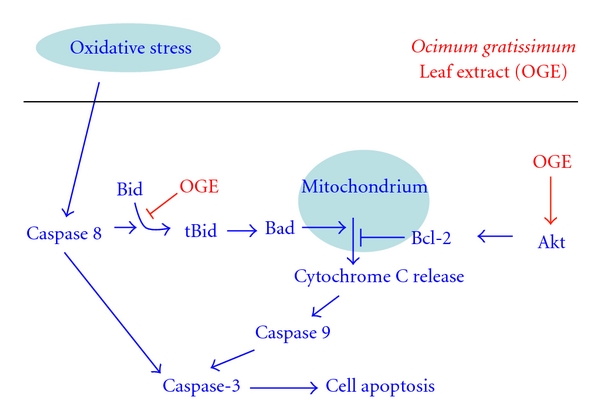
Proposed model for the inhibitory effects of* Ocimum gratissimum* extracts on the oxidative stress-induced cell apoptosis by affecting the mitochondrial pathway. Our data demonstrated that *Ocimum gratissimum* extract attenuates the H_2_O_2_-induced apoptosis of H9c2 myocardiac cells, which may be due to the inhibition of the tBid formation as well as the enhancement of PI3K/Akt survival signaling which includes the increase of antiapoptotic Bcl-2.

Oxidative stress induces cell apoptosis by increasing caspase-3 activation from two mechanisms, as shown in Figure 6. One is the direct activation of caspase-3 by caspase 8, and the other is the activation of caspase-9, also a direct activator of caspase 3, through the mitochondrial pathway. We found that *Ocimum gratissimum* extract helps to prevent cell apoptosis by affecting the mitochondrial pathway in 2 places and that it is able to inhibit the formation of tBid as well as stimulate the activation of Akt, which inhibits the release of CytC through the stimulation of Bcl-2 expression. We also found that *Ocimum gratissimum* extract does not attenuate caspase-8 activation which suggests the alleviation of caspase-8-mediated apoptosis through other mechanisms. Although many polyphenolic acids such as caffeic acid [46] and epigallocatechin gallate [47] have been reported to inhibit the activation of caspase-8, the mechanisms behind OGE's protection of H9c2 cells from H_2_O_2_-induced damage need further investigation. These findings, taken together, indicate that *Ocimum gratissimum* extract may be beneficial in protecting cardiomyocytes from oxidative stress.
